# Hodgkin's lymphoma and infection: findings from a UK case–control study

**DOI:** 10.1038/sj.bjc.6603999

**Published:** 2007-09-25

**Authors:** R Newton, S Crouch, P Ansell, J Simpson, E V Willett, A Smith, C Burton, A Jack, E Roman

**Affiliations:** 1 Department of Health Sciences, Epidemiology and Genetics Unit, University of York, Area 3 Seebohm Rowntree Building, Heslington, York YO10 5DD, UK; 2 Haematological Malignancy Diagnostic Service, Leeds General Infirmary, Leeds, UK

**Keywords:** Hodgkin's, lymphoma, infection

## Abstract

Between 1998 and 2003, 214 people with Hodgkin's lymphoma and 214 controls randomly selected from population registers in the north of England (after matching for age and sex) were recruited and their primary care medical records examined for details of clinical diagnoses due to infectious and non-infectious conditions in the preceding 15 years. In the year before diagnosis of Hodgkin's lymphoma, almost all cases (99%) visited their general practitioner (GP) at least once. In comparison with controls, the excess was evident both for visits with an infection (odd's ratio (OR)=2.1; 95% confidence interval (CI) 1.4–3.2) and for visits with non-infectious problems (OR=17.2; 95% CI 6.7–43.9). During the rest of the 15-year period prior to diagnosis, the proportion of people visiting their GP with a non-infectious condition did not differ between cases and controls. In contrast, compared to controls, there was an excess of cases visiting the GP with an infection, a finding that was evident for at least a decade prior to diagnosis and increased linearly with time (*P*=0.02). This excess was not due to a specific infection(s) and may reflect underlying immune abnormality. Alternatively, infection may cause B-cell proliferation from which a malignant clone may evolve.

The cause(s) of Hodgkin's lymphoma are largely unknown, although various infectious and immune factors have been implicated. A proportion is thought to be related to infection with Epstein Barr virus (EBV), which is integrated clonally into tumour cells in as many as 40% of cases (IARC, 1997). Elevated titres of antibodies against EBV have been associated with subsequent risk of Hodgkin's lymphoma, while infectious mononucleosis, known to be caused by EBV, is an established risk factor (Mueller *et al*, 1989; Hjalgrim *et al*, 2000, 2007). Elevated titres of antibodies against another human herpesvirus (type 6) have been found in some, but not in other studies (Clark *et al*, 1990; Jarrett *et al*, 1998; Berrington de González *et al*, 2006). Infection with human immunodeficiency virus (HIV) may account for a small proportion of cases, although the virus is not thought to have any direct oncogenic activity, but rather to facilitate tumorigenesis via its immune effects (IARC, 1996; Beral and Newton, 1998; Newton *et al*, 1999). Immunosuppression, whether related to HIV infection or drug treatment such as that experienced by transplant recipients, appears to be associated with a modest increase in risk of Hodgkin's lymphoma – substantially less than that of non-Hodgkin's lymphoma (Kinlen *et al*, 1979; Birkeland *et al*, 1995; International Collaboration on HIV and Cancer, 2000; Vajdic *et al*, 2006). No other specific infectious or immune factors have been consistently found.

Unravelling any relationship between risk of Hodgkin's lymphoma and previous infections is not straightforward. To investigate the patterns of infectious illness prior to diagnosis, as well as to identify specific infectious exposures, we systematically abstracted data compiled prior to diagnosis from primary health-care medical records. We report here, on the role of clinically diagnosed infections (as recorded in primary care medical records) in the aetiology of Hodgkin's lymphoma.

## METHODS

Details of the study are described elsewhere (Willett and Roman, 2006; Willett *et al*, 2007). Briefly, cases were patients residing in the north of England between the ages of 16 and 69 years, with newly diagnosed (non-HIV-related) Hodgkin's lymphoma, during 1998–2003. Diagnoses were confirmed pathologically and coded according to the World Health Organisation Classification (http://www.who.int/classifications/icd/adaptations/oncology/en/). For a proportion of cases, EBV status of the tumour was assessed by *in situ* hybridisation for the presence of EBV-EBER in Reed-Sternberg cells (Willett *et al*, 2007). For each case, one control was randomly selected from population registers after matching for age, sex, and region of residence. At interview, cases and controls were asked to consent for their primary care medical records to be accessed by the study team. All information contained within these routinely compiled health records for the 15 years prior to diagnosis in cases (or pseudo-diagnosis in controls) was abstracted onto specially designed forms by trained research staff.

The data collected included all illnesses recorded contemporaneously by the patient's general practitioner (GP) (i.e. their primary care physician), as well as all signs and symptoms recorded at the time, referrals to hospital or other specialists, results of all investigations, and details of medicines or other prescribed therapies. Data collection and entry were structured around dated ‘events’. Disease and drug coding was done centrally by experienced primary care research nurses, using a specially designed computerised system embedded within the data entry programme. Illnesses and symptoms were coded according to the International Statistical Classification of Diseases and Related Health Problems (ICD-10), tenth revision (http://www.who.int/classifications/icd/en/), and drugs to a schema based on the British National Formulary (http://www.bnf.org/bnf/). Strict quality control procedures, including duplicate data entry of a proportion of randomly selected records, were carried out throughout the study period.

Face-to-face interviews were conducted with 284 (79%) of 360 people with Hodgkin's lymphoma diagnosed in the study region and 240 (73%) of 331 controls. Of those, primary care records were accessed for 214 cases and 214 controls – analyses presented here are restricted to these individuals. Ethical approval for the study was granted by the United Kingdom Multi-Regional Ethics Committee.

Analyses were performed using STATA version 9.2 (http://www.stata.com) and R version 2.5.1. (http://www.R-project.org). Data are presented in the form of monthly proportions of patients making at least one visit to their GP for infectious and non-infectious conditions over the 15-year period prior to diagnosis (or pseudo-diagnosis for controls). These proportions were modelled using unconditional logistic regression with time, case–control status, and the time–status interaction as explanatory variables. To prevent the results being skewed by symptoms of disease, events in the 12 months leading to lymphoma diagnosis (pseudo-diagnosis for controls) are excluded. In addition, monthly counts of visits to the GP and of individual diagnoses, were modelled by a negative binomial general linear model (with logarithmic link function), but since the results and conclusions did not materially change, these data are not shown. In disease-specific analyses, odds ratios (OR) and 95% confidence intervals (CI) were estimated using unconditional logistic regression with adjustment for sex and age at diagnosis (in single years).

## RESULTS

Of the 214 cases of Hodgkin's lymphoma, 202 (94%) were characterised as classical Hodgkin's lymphoma (CHL) and 12 (6%) as nodular lymphocyte predominant Hodgkin's lymphoma. Epstein Barr virus status was available for 144 cases, of which 45 (31%) were positive. In the analyses described below, data for classical and nodular lymphocyte predominant Hodgkin's lymphoma are combined. Sixty-three per cent of cases were male and the percentage of cases in age groups 16–29, 30–39, 40–49, and 50–69 was 22, 30, 22, and 26% respectively.

Table 1 shows the number of cases and controls visiting their GP at least once per year for an infectious or non-infectious condition during the 15 years prior to diagnosis of Hodgkin's lymphoma (or pseudo-diagnosis for controls). In all the time periods, about twice as many controls visited at least once a year with a non-infectious condition as compared with an infectious condition. In addition, the proportion of controls visiting a GP at least once increased steadily over time, reflecting the effect of ageing. This trend was driven largely by the proportion visiting for clinical problems other than infections – there was no statistically significant change in the proportion visiting for infection over time.

As expected, in the year before diagnosis of Hodgkin's lymphoma, almost all cases (99%) visited their GP at least once. In comparison with controls, the excess was evident both for visits with an infection (OR=2.1; 95% CI 1.4–3.2) and, more markedly, for visits with other non-infectious problems (OR=17.2; 95% CI 6.7–43.9), including general tiredness and malaise. The total number of visits in the year prior to diagnosis (or pseudo-diagnosis) was 1792 for cases and 824 for controls.

**Figure 1:**
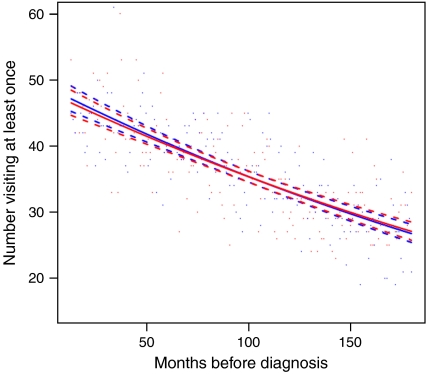
Graph showing the number of cases (in red) and controls (in blue) visiting the general practitioner at least once for a non-infectious condition by individual month for the 15 years prior to diagnosis of Hodgkin's lymphoma, or pseudo-diagnosis in controls (excluding data from the year prior to diagnosis).

**Figure 2:**
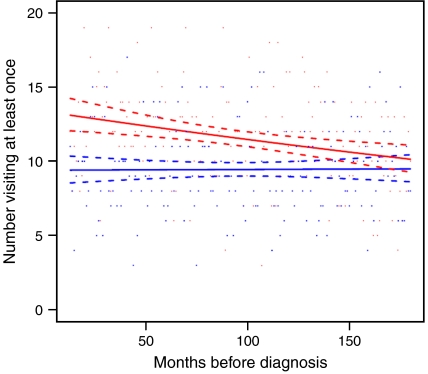
Graph showing the number of cases (in red) and controls (in blue) visiting the general practitioner at least once for an infectious condition by individual month for the 15 years prior to diagnosis of Hodgkin's lymphoma or pseudo-diagnosis in controls (excluding data from the year prior to diagnosis).

Data on the number of patients visiting the GP at least once with an infection or other condition among cases (in red) and controls (in blue) are displayed graphically by individual month for the entire time period (excluding the year prior to diagnosis or pseudo-diagnosis) in Figures 1 and 2. The model predictions are shown in the same figure, along with 95% confidence intervals (dashed lines). In accordance with Table 1, for non-infections (Figure 1), there is a gradual increase in the proportion of cases and controls visiting the GP with advancing age, but no differences between the two groups are evident. In contrast, although the proportion of controls visiting the GP at least once per month with an infection varied little over time (*P*=1.0; Figure 2), for cases, there was an excess, which increased linearly from about 10 years prior to diagnosis (*P*=0.02). This excess among cases as compared to controls did not appear to be due to a specific infection. Data for the most frequently diagnosed infections, together with some that have been linked to Hodgkin's lymphoma in previous studies (such as herpesvirus infections), are shown for the entire time period (excluding the year before diagnosis or pseudo-diagnosis) in Table 2. With the possible exception of herpesviruses and lower respiratory tract infections, no single infection was particularly associated with Hodgkin's lymphoma. The analyses described in Table 2 were repeated, limiting to infections occurring within 10 years prior to diagnosis (but omitting the year before diagnosis) and it made no material difference to the findings (data not shown).

When analyses were restricted to CHL alone, there was no material difference in the results. Similarly, when analyses were stratified by age (<40 and 40+ years), sex, and EBV status of the tumour, the findings remained essentially unchanged (data not shown).

## DISCUSSION

Our analyses of contemporaneously acquired primary care medical data indicate that compared to matched controls, a substantially higher proportion of people who develop Hodgkin's lymphoma visit the GP for an infection, a finding that is evident for at least a decade prior to diagnosis. In contrast, there was little difference between cases and controls in the proportion visiting for a non-infectious problem until about a year before diagnosis when cases showed a marked increase. We cannot, however, determine whether the excess of clinically diagnosed infections is a consequence of underlying immune abnormality or indeed, whether the infections are playing a causal role. No single infection stood out as being specifically associated with Hodgkin's lymphoma and no single event was identified within the 15 years prior to diagnosis that might be associated with disease onset. Although we cannot exclude the possible existence of a single causal agent (possibly acting more than 15 years prior to diagnosis), none was identified.

Large amounts of information on previous illnesses, including infections, are routinely collected by medical practitioners working in primary care. Although these data, which are principally collected with the aim of documenting and monitoring patient care, have been used in a limited way in a number of aetiological studies (McKinney *et al*, 1991; Mann *et al*, 1993; Chilvers *et al*, 1994; Ansell *et al*, 2005), their potential with respect to describing symptom profiles is yet to be fully realised. A critical advantage for aetiological and other studies – where the sequence and timing of events is important – is that information held in GP records is collected prior to the diagnosis of malignancy and so have the advantage of being unaffected by recall and reporting bias, having been recorded by the GP contemporaneously. The methods used for abstracting data from primary care records were originally developed by the investigators (PA and ER). Indeed, the use of clinical records permits a far more precise characterisation of events preceding diagnosis than is possible in studies that rely on self-report (Simpson *et al*, 2007).

As in other epidemiological studies, a fundamental but simplistic distinction is made between Hodgkin's lymphoma and non-Hodgkin's lymphoma which originates in concepts from the early twentieth century. From an epidemiological perspective, Hodgkin's lymphoma appears to be distinctive, with an unusual bimodal age distribution peaking in young adults and in older people (www.hmrn.org). Hodgkin's lymphoma also differs from other types of lymphoma in having a female predominance in the young adult age group. However, to examine the role of infectious and immune factors in relation to Hodgkin's lymphoma stratified by age, sex or indeed by EBV status of the tumour, we would need substantially larger numbers of cases than were available for analysis here. Indeed, in this study as in many others, recruitment was limited to a specific age range (16–69 years), which means, because of the unusual age distribution of Hodgkin's lymphoma, many of the available cases were excluded.

However, it is apparent that CHL is a B-cell malignancy derived from postgerminal centre cells. The defining feature of the tumour cells is the loss of a mature B-cell phenotype but failure to complete differentiation to plasma cells (Cossman *et al*, 1988, 1999; Stein *et al*, 2001; Fan *et al*, 2003). The association of CHL with other B-cell malignancies is emphasised by the occurrence of the so-called composite lymphoma where CHL occurs in association with follicular lymphoma, diffuse large B-cell lymphoma or B-chronic lymphocytic leukaemia, and where both tumours can be shown to have a common clonal origin (Gonzalez *et al*, 1991; Cathcart-Rake *et al*, 1992; Jaffe *et al*, 1992; Kim, 1993; Kuppers *et al*, 2001). Recent gene-expression studies have also shown that at least some cases of CHL have a very similar pattern of gene expression to mediastinal B-cell lymphoma. Both CHL and mediastinal B-cell lymphoma affect the mediastinum and have a female predominance in young adults leading to the suggestion that they are derived from a specific population of thymic B cells (Addis and Isaacson, 1986; Palanisamy *et al*, 2002; Rosenwald *et al*, 2003; Calvo *et al*, 2004). These studies demonstrate the importance of considering CHL in the context of the epidemiology of B-cell malignancies as a whole rather than as a separate entity. Ideally, nodular lymphocyte predominant Hodgkin's lymphoma should be considered separately, although its rarity makes it difficult to recruit the numbers of cases required for epidemiological studies. In the future, high-quality, contemporaneously collected exposure data, together with modern concepts of disease and accurate diagnostic and demographic information, will play a key role in addressing questions of pathogenesis of haematological malignancies.

## Figures and Tables

**Table 1:** Number of cases and controls visiting the general practitioner at least once for an infectious or non-infectious condition during each of the 15 years prior to diagnosis of Hodgkin's lymphoma (or pseudo-diagnosis in controls)

	**Consultations**					
	**All visits**		**Infectious diagnosis**		**Non-infectious diagnosis**	
**Years before diagnosis**	**Controls *N* (%)**	**Cases *N* (%)**	**Controls *N* (%)**	**Cases *N* (%)**	**Controls *N* (%)**	**Cases *N* (%)**
1	164 (77)	212 (99)	69 (32)	106 (50)	153 (72)	209 (98)
2	169 (79)	177 (83)	79 (37)	90 (42)	157 (73)	168 (79)
3	177 (83)	180 (84)	77 (36)	89 (42)	166 (78)	164 (77)
4	160 (75)	180 (84)	67 (31)	92 (43)	146 (68)	166 (78)
5	160 (75)	171 (80)	72 (34)	91 (43)	151 (71)	156 (73)
6	164 (77)	161 (75)	80 (37)	82 (38)	144 (67)	139 (65)
7	164 (77)	159 (74)	70 (33)	78 (36)	147 (69)	148 (69)
8	166 (78)	155 (72)	75 (35)	88 (41)	148 (69)	138 (64)
9	168 (79)	161 (75)	80 (37)	77 (36)	140 (65)	148 (69)
10	164 (77)	155 (72)	67 (31)	91 (43)	151 (71)	134 (63)
11	156 (73)	153 (72)	66 (31)	91 (43)	143 (67)	133 (62)
12	152 (71)	155 (72)	75 (35)	89 (42)	132 (62)	133 (62)
13	144 (67)	157 (73)	76 (36)	72 (34)	123 (57)	143 (67)
14	145 (68)	147 (69)	74 (35)	76 (36)	119 (56)	129 (60)
15	137 (64)	138 (65)	64 (30)	68 (32)	120 (56)	119 (56)

**Table 2:** Number of cases and controls visiting the general practitioner at least once for a specific infection prior to diagnosis of Hodgkin's lymphoma (or pseudo-diagnosis in controls)

**Infections**	**Cases *N* (%)**	**Controls *N* (%)**	**OR (95% CI)**
	214 (100)	214 (100)	
Total^a^	208 (97.2)	199 (93.0)	2.67 (1.01–7.09)
Upper respiratory tract *(J0)*^b^	164 (76.6)	166 (77.6)	0.94 (0.59–1.51)
Skin *(L0)*	81 (37.9)	68 (31.8)	1.31 (0.88–1.95)
Fungal *(B35,B36.0,B37,B49)*	68 (31.8)	67 (31.3)	1.02 (0.67–1.57)
Lower respiratory tract *(J12.9,J18,J20,J21.9,J22)*	62 (29.0)	44 (20.6)	1.58 (1.01–2.48)
Eye *(H01,H10,H05.0)*	61 (28.5)	45 (21.0)	1.50 (0.96–2.34)
Influenza *(J11.1)*	53 (24.8)	48 (22.4)	1.14 (0.73–1.78)
			
*Herpes*	42 (19.6)	21 (9.8)	2.33 (1.31–4.14)
-Simplex *(A60.0,B00)*	16 (7.5)	9 (4.2)	1.87 (0.80–4.38)
—Varicella/Zoster *(B01.9,B02.9)*	15 (7.0)	11 (5.1)	1.39 (0.62–3.11)
-Glandular fever/EBV *(B27.0,B27.9)*	6 (2.8)	2 (0.9)	3.13 (0.62–15.90)
			
Ear *(H66.9)*	34 (15.9)	37 (17.3)	0.90 (0.54–1.51)
Urinary tract *(N39.0)*	26 (12.2)	20 (9.4)	1.36 (0.72–2.55)
Sexually transmitted *(A54.9,A56.2,A59.9,A63.0,A64)*	5 (2.3)	7 (3.3)	0.70 (0.22–2.28)

CI=confidence interval; EBV=Epstein Barr virus; OR=odd's ratio.

a Includes *ICD-10*, codes A00-B99, H01, H05.0, H10, H66.9, L0, L42, J0–J2 & N39.0.

b Data in parentheses *ICD-10* code(s).

## References

[bib1] Addis BJ, Isaacson PG (1986) Large cell lymphoma of the mediastinum: a B-cell tumour of probable thymic origin. Histopathology 10: 379–390 2423430 10.1111/j.1365-2559.1986.tb02491.x

[bib2] Ansell P, Mitchell CD, Roman E, Simpson J, Birch JM, Eden OB (2005) Relationships between perinatal and maternal characteristics and childhood hepatic tumours. A report from the UKCCS. Eur J Cancer 41(5): 741–748 15763651 10.1016/j.ejca.2004.10.024

[bib3] Beral V, Newton R (1998) Overview of the epidemiology of immunodeficiency associated cancers. J Natl Cancer Inst Monogr 23: 1–6 10.1093/oxfordjournals.jncimonographs.a024164 9709294

[bib4] Berrington de González A, Urban M, Sitas F, Blackburn N, Hale M, Patel M, Ruff P, Sur R, Newton R, Beral V (2006) Antibodies against six human herpesviruses in relation to seven cancers in black South Africans: a case control study. Infect Agent Cancer 1: 2 (available on-line) 17150131 10.1186/1750-9378-1-2 PMC1635002

[bib5] Birkeland SA, Storm HH, Lamm LU, Barlow L, Blohme I, Forsberg B, Eklund B, Fjeldborg O, Friedberg M, Frõdin L (1995) Cancer risk after renal transplantation in the Nordic countries, 1964–1986. Int J Cancer 60: 183–189 7829213 10.1002/ijc.2910600209

[bib6] Calvo KR, Traverse-Glehen A, Pittaluga S, Jaffe ES (2004) Molecular profiling provides evidence of primary mediastinal large B-cell lymphoma as a distinct entity related to classic Hodgkin lymphoma: implications for mediastinal gray zone lymphomas as an intermediate form of B-cell lymphoma. Adv Anat Pathol 11: 227–238 15322489 10.1097/01.pap.0000138144.11635.f8

[bib7] Cathcart-Rake WF, Macy NE, Stephens RL (1992) True composite lymphoma: non-Hodgkin's lymphoma and Hodgkin's disease. Kans Med 93: 151–154 1619839

[bib8] Chilvers CE, Pike MC, Taylor CN, Hermon C, Crossley B, Smith SJ (1994) General practitioner notes as a source of information for case–control studies in young women. UK national case–control study group. J Epidemiol Community Health 48: 92–97 8138777 10.1136/jech.48.1.92 PMC1059901

[bib9] Clark DA, Alexander FE, McKinney PA, Roberts BE, O'Brien C, Jarrett RF, Cartwright RA, Onions DE (1990) The seroepidemiology of human herpesvirus-6 (HHV-6) from a case–control study of leukaemia and lymphoma. Int J Cancer 45(5): 829–833 2159435 10.1002/ijc.2910450507

[bib10] Cossman J, Annunziata CM, Barash S, Staudt L, Dillon P, He WW, Ricciardi-Castagnoli P, Rosen CA, Carter KC (1999) Reed-Sternberg cell genome expression supports a B-cell lineage. Blood 94: 411–416 10397707

[bib11] Cossman J, Sundeen J, Uppenkamp M, Sussman E, Wahl L, Coupland R, Lipford E, Raffeld M (1988) Rearranging antigen-receptor genes in enriched Reed-Sternberg cell fractions of Hodgkin's disease. Hematol Oncol 6: 205–211 3402916 10.1002/hon.2900060303

[bib12] Fan Z, Natkunam Y, Bair E, Tibshirani R, Warnke RA (2003) Characterization of variant patterns of nodular lymphocyte predominant hodgkin lymphoma with immunohistologic and clinical correlation. Am J Surg Pathol 27: 1346–1356 14508396 10.1097/00000478-200310000-00007

[bib13] Gonzalez CL, Medeiros LJ, Jaffe ES (1991) Composite lymphoma. A clinicopathologic analysis of nine patients with Hodgkin's disease and B-cell non-Hodgkin's lymphoma. Am J Clin Pathol 96: 81–89 2069139 10.1093/ajcp/96.1.81

[bib14] Hjalgrim H, Askling J, Sørenson P, Madsen M, Rosdahl N, Storm HH, Hamilton-Dutoit S, Eriksen LS, Frisch M, Ekbom A, Melbye M (2000) Risk of Hodgkin's disease and other cancers after infectious mononucleosis. J Natl Cancer Inst 92(18): 1522–1528 10995808 10.1093/jnci/92.18.1522

[bib15] Hjalgrim H, Smedby KE, Rostgaard K, Molin D, Hamilton-Dutoit S, Chang ET, Ralfkiaer E, Sundstrõm C, Adami HO, Glimelius B, Melbye M (2007) Infectious mononucleosis, childhood social environment and risk of Hodgkin lymphoma. Cancer Res 67(5): 2382–2388 17332371 10.1158/0008-5472.CAN-06-3566

[bib16] IARC (1997) IARC monograph on the evaluation of carcinogenic risks to Humans. Epstein-Barr Virus and Kaposi's Sarcoma Herpesvirus/Human Herpesvirus 8, vol. 67 Lyon: IARC PMC5046075 9705682

[bib17] IARC (1996) IARC monograph on the evaluation of carcinogenic risks to Humans. Human Immunodeficiency Viruses And Human T-Cell Lymphotropic Viruses, vol. 67 Lyon, France: IARC PMC5366879 9190379

[bib18] International Collaboration on HIV and Cancer (2000) The impact of highly active anti-retroviral therapy on the incidence of cancer in people infected with the Human Immunodeficiency Virus. J Natl Cancer Inst 92: 1823–1830 11078759 10.1093/jnci/92.22.1823

[bib19] Jaffe ES, Zarate-Osorno A, Medeiros LJ (1992) The interrelationship of Hodgkin's disease and non-Hodgkin's lymphomas—lessons learned from composite and sequential malignancies. Semin Diagn Pathol 9: 297–303 1480852

[bib20] Jarrett RF, Gledhill S, Qureshi F, Crae SH, Madhok R, Brown I, Evans I, Krajewski A, O'Brien CJ, Cartwright RA (1998) Identification of human herpesvirus 6-specific DNA sequences in two patients with non-Hodgkin lymphoma. Leukemia 2(8): 496–502 3412023

[bib21] Kim H (1993) Composite lymphoma and related disorders. Am J Clin Pathol 99: 445–451 8475911 10.1093/ajcp/99.4.445

[bib22] Kinlen LJ, Sheil AGR, Peto J, Doll R (1979) Collaborative United Kingdom–Australasian study of cancer in patients treated with immunosuppressive drugs. BMJ 2: 1461–1466 393355 10.1136/bmj.2.6203.1461 PMC1597175

[bib23] Kuppers R, Sousa AB, Baur AS, Strickler JG, Rajewsky K, Hansmann ML (2001) Common germinal-center B-cell origin of the malignant cells in two composite lymphomas, involving classical Hodgkin's disease and either follicular lymphoma or B-CLL. Mol Med 7: 285–292 11474574 PMC1950043

[bib24] Mann JR, Dodd HE, Draper GJ, Waterhouse JA, Birch JM, Cartwright RA, Hartley AL, McKinney PA, Stiller CA (1993) Congenital abnormalities in children with brain tumours and their relatives: results from a case–control study. Br J Cancer 68: 357–363 8347491 10.1038/bjc.1993.340 PMC1968541

[bib25] McKinney PA, Alexander FE, Nicholson C, Cartwright RA, Carrette J (1991) Mothers' reports of childhood vaccinations and infections and their concordance with general practitioner records. J Public Health Med 13: 13–22 2029431 10.1093/oxfordjournals.pubmed.a042571

[bib26] Mueller N, Evans A, Harris NL, Comstock GW, Jellum E, Magnus K, Orentreich N, Polk BF, Vogelman J (1989) Hodgkin's disease and epstein-barr virus. Altered antibody pattern before diagnosis. N Engl J Med 320(11): 689–695 2537928 10.1056/NEJM198903163201103

[bib27] Newton R, Beral V, Weiss R (1999) Human immunodeficiency virus infection and cancer. In Cancer Surveys Volume 33, Infections and Human Cancer. Newton R, Beral V, Weiss R (eds) Cold Spring Harbor Laboratory Press: USA

[bib28] Palanisamy N, Abou-Elella AA, Chaganti SR, Houldsworth J, Offit K, Louie DC, Terayu-Feldstein J, Cigudosa JC, Rao PH, Sanger WG, Weisenburger DD, Chaganti RS (2002) Similar patterns of genomic alterations characterize primary mediastinal large-B-cell lymphoma and diffuse large-B-cell lymphoma. Genes Chromosomes Cancer 33: 114–122 11793437 10.1002/gcc.10016

[bib29] Rosenwald A, Wright G, Leroy K, Yu X, Gaulard P, Gascoyne RD, Chan WC, Zhao T, Haioun C, Greiner TC, Weisenburger DD, Lynch JC, Vose J, Armitage JO, Smeland EB, Kvaloy S, Holte H, Delabie J, Campo E, Montserrat E, Lopez-Guillermo A, Ott G, Muller-Hermelink HK, Connors JM, Braziel R, Grogan TM, Fisher RI, Miller TP, LeBlanc M, Chiorazzi M, Zhao H, Yang L, Powell J, Wilson WH, Jaffe ES, Simon R, Klausner RD, Staudt LM (2003) Molecular diagnosis of primary mediastinal B cell lymphoma identifies a clinically favorable subgroup of diffuse large B cell lymphoma related to Hodgkin lymphoma. J Exp Med 198: 851–862 12975453 10.1084/jem.20031074 PMC2194208

[bib30] Simpson J, Smith A, Ansell P, Lightfoot T, Hughes A-M (2007) Roman E on behalf of the United kingdom childhood cancer study investigators. Childhood leukaemia and markers of infectious exposure: a report from the United Kingdom Childhood Cancer Study (UKCCS). Eur J Cancer, doi:10.1016/j.ejca.2007.07.027 10.1016/j.ejca.2007.07.027 17826085

[bib31] Stein H, Marafioti T, Foss HD, Laumen H, Hummel M, Anagnostopoulos I, Wirth T, Demel G, Falini B (2001) Down-regulation of BOB.1/OBF.1 and Oct2 in classical Hodgkin disease but not in lymphocyte predominant Hodgkin disease correlates with immunoglobulin transcription. Blood 97: 496–501 11154228 10.1182/blood.v97.2.496

[bib32] Vajdic CM, McDonald SP, McCredie MR, van Leeuwen MT, Stewart JH, Law M, Chapman JR, Webster AC, Kaldor JM, Grulich AE (2006) Cancer incidence before and after kidney transplantation. JAMA 296(23): 2823–2832 17179459 10.1001/jama.296.23.2823

[bib33] Willett EV, O'Connor S, Smith AG, Roman E (2007) Does smoking and alcohol modify the risk of Epstein-Barr virus-positive or -negative Hodgkin lymphoma. Epidemiology 18(1): 130–136 17099321 10.1097/01.ede.0000248899.47399.78

[bib34] Willett EV, Roman E (2006) Obesity and the risk of Hodgkin lymphoma (United Kingdom). Cancer Causes Control 17(8): 1103–1106 16933061 10.1007/s10552-006-0042-6

